# NOTHING FOR ME WITHOUT ME: CAREGIVER TRAINING NEEDS

**DOI:** 10.1093/geroni/igz038.1419

**Published:** 2019-11-08

**Authors:** Jeananne Elkins, Phillip Rustin

**Affiliations:** Brenau University, Gainesville, Georgia, United States

## Abstract

Over 34 million informal caregivers provide care to an adult aged 50 or older with over 15 million caregivers caring for a person with dementia. People often become caregivers unexpectedly. For most caregivers little training is available, and most skills are learned “on the job”. The objective of this study is to determine the training needs of caregivers. An anonymous survey was conducted at two caregiver conferences. Caregivers were asked to name up to 5 training needs. For this abstract we report the percent of responses by caregivers. Seventy-nine caregivers completed the survey. Seventy-seven percent (77%) of the caregivers cared for a person over age 50. Increased knowledge of health issues (17%) and resources (17%) were the highest responses. Training in physical caregiving skills i.e. bathing (10%), transfers (8%) and diaper changes (4%) were voiced as important. Training for improving personal interactions with the care recipient was important (9%) as well as how to practice patience, kindness and compassion (7%). Two areas where training is available but perhaps not accessible – caregiver support (9%) and care recipient behaviors (8%) were all important to caregivers. Healthy eating (9%) and stopping or limiting driving by the care recipient (2%) were enunciated as areas requiring training. Many of the training needs should be addressed by healthcare professionals in their interactions with caregivers and care recipients. Programs are available online, but caregivers are not accessing this training. To improve outcomes for both caregivers and care recipients targeted training for caregivers is needed.

